# Variation in thyroid hormone levels is associated with elevated blood mercury levels among artisanal small-scale miners in Ghana

**DOI:** 10.1371/journal.pone.0203335

**Published:** 2018-08-30

**Authors:** Justice Afrifa, Wisdom Djange Ogbordjor, Ruth Duku-Takyi

**Affiliations:** 1 Department of Medical Laboratory Science, School of Allied Health Sciences, University of Cape Coast-Cape Coast, Ghana; 2 Volta River Authority, Accra, Ghana; Stony Brook University, Graduate Program in Public Health, UNITED STATES

## Abstract

**Background:**

Mercury can be very toxic to human health even at low dose of exposure. Artisanal small-scale miners (ASGMs) use mercury in gold production, hence are at risk of mercury-induced organ dysfunction.

**Aim:**

We determined the association between mercury exposure, thyroid function and work-related factors among artisanal small-scale gold miners in Bibiani- Ghana.

**Method:**

We conveniently recruited 137 consenting male gold miners at their work site in Bibiani-Ghana, in a comparative cross-sectional study. Occupational activities and socio-demographic data of participants were collected using a questionnaire. Blood sample was analysed for total mercury and thyroid hormones.

**Results:**

Overall, 58.4% (80/137) of the participants had blood mercury exceeding the occupational exposure threshold (blood mercury ≥5μg/L). T3(P<0.0001) and T4(P<0.0001) were significantly reduced among the exposed group compared to the non-exposed. TSH showed no significant variation between the exposed and non-exposed groups. Longer work duration (≥5years), gold amalgamation, gold smelting and sucking of excess mercury with the mouth were associated with increased odds of mercury exposure. Blood mercury showed negative correlation with T3(r = -0.29, P<0.0001), and T4(r = -0.69, P<0.0001) and positive correlation with work duration (r = 0.88, P<0.001). Even though a positive trend of association between blood mercury and TSH levels was recorded, it was not significant (r = 0.07, P = 0.4121)

**Conclusion:**

Small scale miners in Bibiani are exposed to mercury above the occupational threshold which may affect thyroid hormone levels.

## Introduction

Artisanal or small-scale mining by definition are those operations using only rudimentary or artisanal implements as well as more sophisticated mining activities operating at a relatively low level of production and which generally require limited capital investment [1]. Artisanal small-scale mining is largely a poverty driven activity that constitutes an important source of livelihood for many rural communities, but it is also the world’s fastest growing source of mercury contamination [2, 3]. Mining methods that are employed by these small-scale miners of precious minerals vary according to the type of deposit being exploited and its location [1]. Mercury amalgamation serves as one of the major tools employed in extraction of gold among small-scale miners [4].

Mercury (Hg) is a global threat to human health with severe impact on physiological processes in the human body [5]. Environmental contamination of mercury from old mines as well as on going mining practices which significantly rely on mercury amalgamation in the extraction of gold is common. Such contaminations are known to impact negatively on various environmental systems at varying levels [6]. Among humans, mercury causes alteration in the physiological and the biochemical functions which present with a wide range of clinical symptoms [7]. Mercury has adverse effects on a variety of systems that vary with the level, length of exposure, exposure duration and the form of mercury being exposed [5]. The toxicity of mercury primarily targets the nervous system, however depending on the specific type of mercury and extent of exposure, the kidneys, liver, lungs and thyroids are also important targets [8, 9].

At low concentrations, mercury is able to inhibit the biosynthesis of thioredoxin reductase and other selenoenzymes through the depletion of cellular selenium; hence, inhibiting reversal of oxidative damage [10]. The mechanisms of mercury related thyroid hormone disruption involve selective binding to sulfhydryl containing ligands in the thyroid, disruptions in thyroid stimulating hormone production, and inhibition of deiodination [11]. Hence, a possible elevation in blood mercury concentrations above occupational threshold may lead to changes in thyroid hormone levels among artisanal small-scale miners at Bibiani.

Mercury threatens human health and can impair many physiological processes. Recent studies suggest that particularly small-scale miners are vulnerable to levels of mercury commonly found in their environments and work place. In Ghana, small-scale miners use mercury for mining and have direct contact with mercury daily. Most of these miners work with mercury without enough protection and education on the safe use of mercury. However emerging studies have tried to shed light on some of the negative consequences of small-scale mining from different perspectives [12–14]. These include studies geared towards regulating ASGM practices [12], ASGM and living conditions as well as effect of ASGM on neurotoxic health risk [13]. Also, we have previously reported a reduced estimated glomerular filtration rate, elevated urine protein and low level of personal protective equipment compliance among artisanal small-scale gold miners at Bibiani in Ghana [14]. Our current study is the first to report on the association of blood mercury levels with thyroid hormone variations among small-scale gold miners in the Sub-Saharan Africa region.

## Materials and methods

### Study area

This comparative cross-sectional study was carried out, between January, 2017 and March, 2018 at Bibiani an old gold mining town in the Bibiani -Anhwiaso—Berkwai district of the Western region of Ghana. Bibiani, the district capital is 356km from Sekondi-Takoradi, the regional capital and 88km from Kumasi in the Ashanti Region. There are two small scale-mining sites in Bibiani namely Donkoto and Zongo sites respectively and a large gold mining company namely Mensin Gold Mining Company in the town.

### Study population and sampling

A purposive sampling was used to recruit 137 male participants in January 2017, who were actively involved in ASGM at the time of the study.

### Inclusion/exclusion criteria

The small-scale miners were selected for the study based on the following criteria: at least 1year contact with mercury in the mines. Small-scale miners that failed to give an informed consent and persons not engaging in small-scale gold mining at Bibiani were excluded from the study. Small-scale miners that had previously worked in other areas that is likely to expose them to lead, cadmium and other hepatotoxic substances were excluded from the study. Subjects with known active (acute or chronic) liver diseases such as hepatitis, alcohol liver disease, drug induced liver disease as well as those with known thyroid diseases were excluded from the study.

### Ethical approval and consent to participate

The institutional review board of the University of Cape Coast gave their approval for the commencement of the study (Ethical Clearance ID: UCCIRB/CHAS/2016/61). Participants were fully informed about the purpose, procedures, risks, and benefits of participating in this study. Written informed consent was sought from the participants before recruitment.

### Data collection tool

A pre-tested structured questionnaire was administered to the 137 consenting participants to collect their socio-demographics characteristics, and work site related activities which may lead to increased mercury exposure.

### Blood sample collection and processing

Five (5) ml of blood sample was collected from each participant with the aid of sterilized syringes and needles. Three (3) ml of the blood sample was put into EDTA K_3_ tubes and the rest into serum separator tubes. Samples were kept inside a cold plastic box containing ice packs in the field. All the samples were transported to the laboratory at Bibiani district hospital. Blood samples in the serum separator tubes were centrifuged at a speed of 1000rpm for 10minutes to obtain the serum, adequately labelled and stored at -20°C. Samples were then kept in ice packs and were transported to the Volta River Authority hospital (VRA), Akosombo for thyroid hormone analyses. After digestion, blood samples were transported to the Ghana Atomic Energy Commission (GAEC) in Accra for mercury analysis. Thyroid hormones; (triiodothyronine(T3), thyroxine(T4) and Thyroid stimulating hormone(TSH) were analyzed at the VRA hospital.

### Blood mercury analysis (wet digestion)

The method for blood mercury analysis was similar to the procedure reported in our earlier study [14]. Wet digestion of blood mercury was performed at research the laboratory in the School of Agricultural of the University of Cape Coast. Briefly, 2g of blood sample was transferred into the bottom of a thick-walled volumetric digestion flask, 6 ml of conc. HNO_3,_ and 1 ml of 30% H_2_O_2_ were added in turn and the mixture was heated at a temperature of 45°C inside a block digester (model: EFA-5UDRVW-8) for 3 hours. The mixture was allowed to cool and distilled water was added to make a fixed volume of 30ml, it was well mixed, and the resulting solution was used as the sample test solution. Samples were kept inside mercury free plastic containers and transported to Ghana Atomic Energy Commission (GAEC) in Accra for mercury analysis. The cold vapour atomic absorption spectrophotometer (CVAAS), (varian model AA240FS), with a detection limit of 0.01ug/L was used for the analysis. The certified reference materials(CRM) used was obtained from TraceCERT®, SIGMA-ALDRICH®, USA. Individuals with blood mercury levels ≥5.0 μg/L were considered as occupationally exposed and those with mercury levels <5.0μg/L were considered as non-exposed [15, 16]

### T4, T3 and TSH

Serum T3, T4 and TSH were estimated using the Ichroma^TM^ II reader at the VRA hospital.

### Data analysis

Data were analyzed using Graph pad prism version 6.01, SPSS version 22 and R statistical software package. Exploratory analysis was carried out to obtain descriptive statistics such as frequencies, percentages, Mean ± standard deviation, figures and tables. Mann-Whitney U test was used to compare the median values of work duration, age and levels of blood mercury concentration of exposed and non-exposed participants respectively and data presented as median (interquartile range). Student sample t-test was performed to compare exposed and non-exposed for T3, T4 and TSH. Fisher’s exact test or Chi-square test where appropriate was used to estimate association between proportions of variables in exposed and non-exposed group. Crude Odds Ratio and 95% CI were reported for Fischer exact test outcome. Spearman rho moment correlation analysis was performed to determine correlation between mercury concentration and serum T3, T4, TSH levels and work duration of occupationally exposed participants. Significance level was determined at *P*< 0.05.

## Results

The most represented age group was 21–39 years for both exposed 73.8%(59/80) and non-exposed 59.7%(34/57) participants. There was no significant difference between median age of exposed (26 years) and non-exposed (30 years) participants (P = 0.0949). The majority of both exposed 81.3% (65/80) and non-exposed 68.4% (39/57) participants were married. In general, about 46.7%(63/137) of the total participants had no formal education whiles 27.7%(38/137) had completed secondary education. The median work duration (P<0.001) and blood mercury levels (P<0.001) were significantly higher among exposed group compared to the non-exposed group. Levels of T3 (P<0.001), and T4 (P<0.001) were significantly reduced among the exposed group as compared to the non-exposed group. However, TSH (P = 0.7749), did not show any significant difference between the exposed and non-exposed participants (Table 1).

**Table 1:** Socio-demographic characteristics, thyroid function markers and blood mercury concentrations of study participants stratified by mercury levels above occupational threshold.

Variable	Exposed N = 80(58.4)	Non-Exposed N = 57(41.6)	P-value
**Age(years)**	26(22.25–35.75)	30(24.0–39.0)	0.0949^M^
≤20	6(7.5)	10(17.5)	0.0005^**C**^
21–39	59(73.8)	34(59.7)	
40–59	5(6.3)	13(22.8)	
≥60	9(11.3)	0(0.00)	
***Marital status***			0.1054^**C**^
Married	65(81.3)	39(68.4)	
Single	15(18.7)	18(31.6)	
***Highest level of education***			0.0148^**C**^
None	29(36.25)	34(59.65)	
Primary	29(36.25)	7(12.28)	
Secondary	22(27.5)	16(28.07)	
***Work duration (Years)***	8.0(7.0–9.0)	4.0(3.0–4.0)	<0.0001^M^
≥5	74(92.50)	8(14.04)	<0.0001^**C**^
<5	6(7.50)	49(85.96)	
T3(nmol/L)	1.54 ± 0.93	2.46 ± 1.47	<0.0001^**t**^
T4(ug/dl)	5.44 ± 1.36	9.6 ± 2.87	<0.0001^**t**^
TSH(mIU/L)	1.70 ± 0.54	1.67 ± 0.90	0.7749^**t**^
Blood Mercury(ug/L)	8.0(6.0–9.0)	1.0(1.0–3.0)	<0.0001^M^

Values are presented as frequency (percentages), Mean± SD, Median (IQR)

^M^Mann Whitney U-test;

^t^student sample t-test;

^c^chi-square test

**Fig 1:**
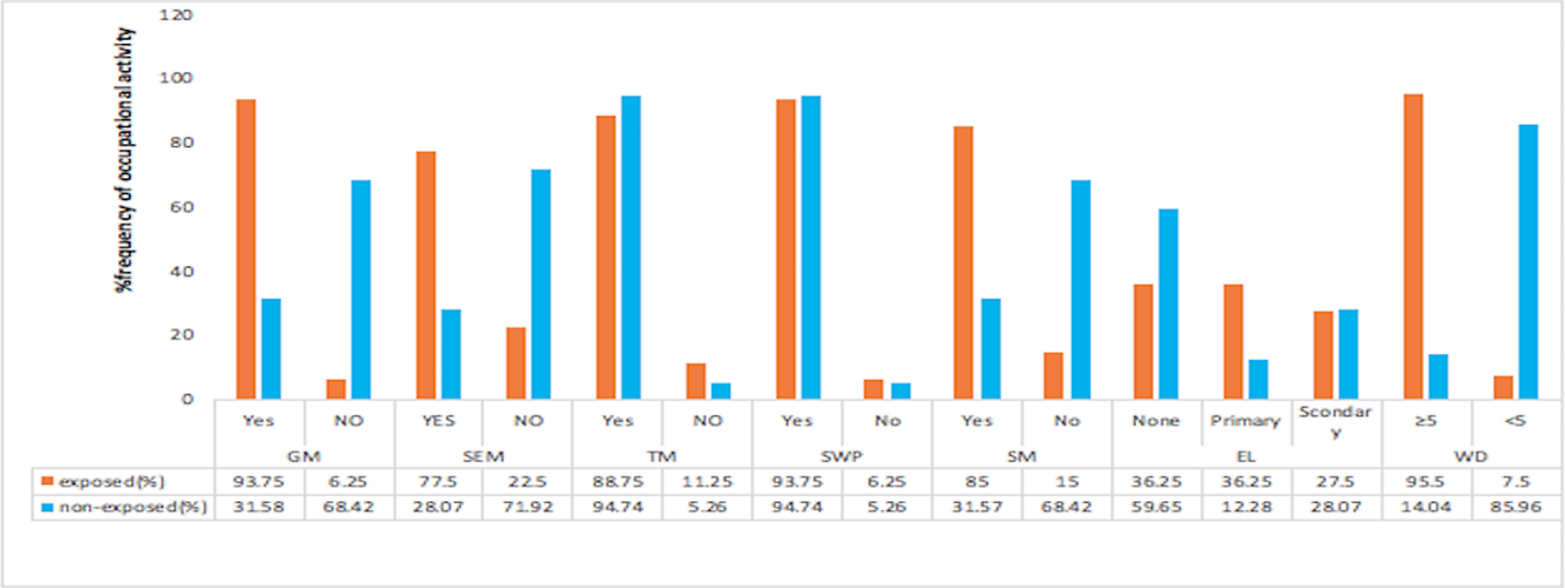
Percentage frequencies of occupational activities among small scale miners stratified by mercury exposure. GM- Gold amalgamation, SEM- sucking of excess mercury, TM- transport of mercury, SWP- standing in water pool, SM- smelting of gold, EL- Educational level, WD-work duration.

A higher percentage of the exposed participants were involved in gold amalgamation (93.76%), sucking of excess mercury (77.5%), smelting of gold (85%) and longer work duration above 5years (95.5%). On the contrary majority of the participants that were involved in activities such as transporting mercury (94.74%) and standing in water pool (94.74%) were not exposed to mercury above 5ug/l (Fig 1).

In the assessment of occupational activities associated with mercury exposure, gold amalgamation [OR = 32.5(95%CI = 11.23–81.89), P<0.001], gold smelting [OR = 8.83 (95%CI = 3.94–19.08), P<0.001], sucking of excess mercury [OR = 12.28(95%CI = 5.41–26.89) P<0.0001] and work duration of 5 years and above [OR = 75.54(23.09–208.4), P<0.001] were significantly associated mercury exposure (Table 2).

**Table 2:** Association of various work-related factors and educational status with mercury exposure.

	Mercury Exposure			
Variable	Exposed ≥5ug/dl	Non-exposed <5ug/dl	Odds Ratio(95%CI)	P-value
**Gold amalgamation**				
Yes	75	18	32.5(11.23–81.89)	<0.0001
No	5	39	*	
Gold smelting				
Yes	62	16	8.83 (3.94–19.08)	<0.0001
No	18	41	*	
Mercury transport				
Yes	71	54	0.44(0.12–1.75)	0.3586
No	9	3		
Standing in water pool				
Yes	75	54	0.83(0.21–3.36)	>0.9999
No	5	3	*	
Sucking of excess mercury				
Yes	68	18	12.28(5.41–26.89)	<0.0001
No	12	39	*	
Educational Level				
None	29	34		
Primary	29	7	0.21(0.08–0.53)	0.0013
Secondary	22	16	0.62(0.28–1.44)	0.3059
Work Duration			*	
≥5	74	8	75.54(23.09–208.4)	<0.0001
<5	6	49	*	

*reference variable

**Fig 2:**
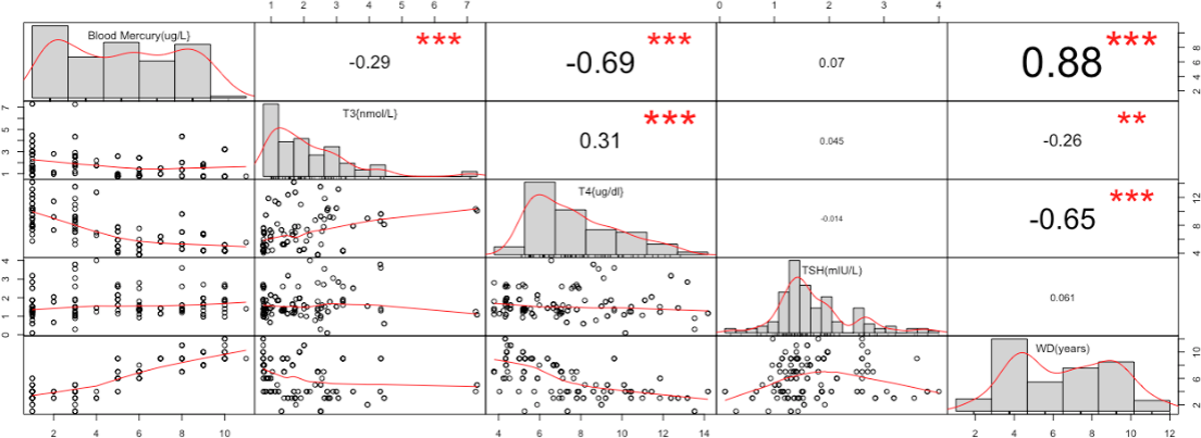
Scatter plot and correlation matrix among blood mercury, work duration, T3, T4 and TSH among study participants. ** indicates significant correlation numbers show the correlation coefficient.

Correlation analysis between blood mercury concentration, work duration, T3, T4 and TSH indicated that, T3(r = -0.29, P<0.0001), and T4(r = -0.69, P<0.0001) showed a negative correlation with elevated blood mercury levels. There was a significant positive correlation between work duration (r = 0.88, P<0.0001) and blood mercury levels. However, TSH (r = 0.07, P = 0.4121) levels even though positively correlated with blood mercury was not statistically significant. Also, T3 correlated positively with T4 whiles duration of work correlated negatively with both T3 and T4 (Fig 2).

## Discussion

Thyroid hormones classically function to regulate metabolism, growth, reproduction, cellular differentiation as well as mediating cellular response to both exogenous and endogenous stimuli [17]. Therefore, disruption of thyroid function is very detrimental to human health. Earlier studies have implicated heavy metals such as mercury in the disruption of the hypothalamus-pituitary- thyroid (HPT) system [18]. With artisanal small-scale mining being one of the major contributors of environmental mercury contamination world-wide [19–21] as well as the direct contact of artisanal small-scale miners with mercury through various occupational activities, we sought to assess thyroid hormone levels among small scale gold miners at the Bibiani-Awiaso district, in the Western region of Ghana.

Our results indicate that overall, 58.4% (80/137) of participants had blood mercury levels exceeding the occupational exposure threshold (blood mercury ≥5μg/L). T3(P<0.0001) and T4(P<0.0001) were significantly reduced among the exposed group compared to the non-exposed, however even though TSH was slightly elevated among the exposed group, it was not significant(P = 0.5913). It must be noted that, even though both T3 and T4 were significantly reduced among the exposed group compared to the non-exposed group, the recorded means for both groups were within the normal range for adults and thus may not necessarily lead to immediate thyroid diseases among these people. We also report a significantly negative correlation between blood mercury levels and both T3(r = -0.29, P<0.0001) and T4(r = -0.69, P<0.0001) levels. Even though a positive trend of association was recorded between blood mercury levels and TSH levels, it was not significant (r = 0.07, P = 0.4121). Our results also indicated that participants who have worked in the mines for a longer period of time(≥5years) had correlated increase in blood mercury levels (r = 0.88, P<0.0001). Longer work duration, gold amalgamation, gold smelting and sucking of excess mercury with the mouth were associated with increased odds of mercury exposure.

Occupational mercury exposure levels have been linked to several factors including the number of years of exposure, type and nature of mercury and state of mercury [22]. In line with our current data, previous studies conducted in South Africa [23], Venezuela [24] and Ghana [14, 25] have demonstrated that a high percentage of small scale miners are exposed to mercury above the occupational threshold. Mercury toxicity is characteristically insidious [26] which supports our finding that extended work duration is associated with increased blood mercury level among small-scale miners.

Mercury has been implicated in the inhibition of one or more specific enzymes along the pathways in the biosynthesis of hormones through its ability to reduce hormone receptor and as a result might impair endocrine function [27]. The thyroid gland like the pituitary displays high affinity for mercury accumulation [28]. Even low levels of mercury may lead to thyroid inflammation, body temperature impairment, hypothyroidism, and depression by occupying iodine binding site and inhibiting or altering hormone action [29, 30]. Our results show a decrease in T4 and T3 with an increasing blood mercury levels. This is in line with a an earlier study which reported that the effect of mercury on organ damage may cause a transient decrease in serum T4 levels followed by an increase in serum TSH levels [31].

Usually a well-functioning status of the thyroid is assessed by measuring both free and bound fractions of T4 and T3 which derive it optimal sustainability through pituitary feedback mechanisms. Deiodination serves as the major route for T4 and T3 metabolism and contributes to 50% of T3 metabolism[32]. It can be both an activating mechanism (production of T3) and an inactivating mechanism [production of rT3 or T2 (di-iodothyronine)][33]. Kawada et al.,(1980) explains that, methyl mercury causes a coupling defect in the synthesis of iodothyronines [34], and in other studies methyl mercury was shown to be concentrated in the thyroidal cells[35]. The accumulation of mercury in the exposed group might explain the significantly reduced circulatory T4. This will further reduce the uptake of the T4 by the liver cells from the circulatory blood and thus significantly decrease the circulatory T3.

Another plausible explanation for the decreased T3 among mercury exposed group is that, mercury primarily targets the liver and this may inhibit 5'- thyroxine deiodinase type II, which is involved in the conversion of T4 to T3. In fact, studies have demonstrated that, mercury exposed mice developed a conspicuously damaged and degenerative liver tissue with necrotic changes as well as significant increase in serum ALT and AST and a decrease in serum ALP [36]. There is abundant evidence of mercury mediated deiodinase activity inhibition in adult rat liver[37–39].

We report a slightly but insignificant elevated serum TSH among exposed group compared to the non-exposed group and this is supported by a positive but insignificant correlation between mercury levels and TSH. This may be due to an initial but uncompensated feedback mechanism resulting from the relatively low T4 and T3. TSH function as the principal stimulator of thyroid hormones from the anterior pituitary and synthesis of major functional players including iodide transporter, thyroid peroxidase and thyroglobulin synthesis are enhanced when TSH binds to receptors on thyroid epithelial cell.

As previously stated, mercury is insidious and toxic even at very low dose of exposure[22]. This may ensure sublime toxicity through several routes including skin contacts, oral ingestions as well as nasal inhalations. However, at room temperature, ingestion of liquid mercury may pose minimal threat due to non-absorption through the gastrointestinal tract. On the contrary vaporous mercury presents as the main route of toxic exposure. This may explain why activities mostly related to heating and burning of mercury such as gold smelting and amalgamation were significantly associated with increased blood mercury levels. Risher and Amler, (2005) report that, the main route of mercury exposure among miners is through inhalation of significant concentration of mercury vapor during processing and extraction of gold[40]. Current reports indicates that the use of urine mercury will be a better indicator for chronic as well as inorganic mercury exposure compared to blood mercury which contains a higher percentage of methyl mercury and poses some limitations on our current study, however studies[41, 42] have demonstrated a correlation between blood mercury levels and mercury concentration in the urine. We also acknowledge the absence of pure control groups who are not in direct contact with mercury in the line of their occupation which would have added extra stability to our data and conclusions.

## Conclusion

Small scale miners in Biabiani are exposed to mercury above the occupational threshold which may affect thyroid hormone levels. However, the mean T3 and T4 levels measured in our study population were within the normal ranges for adults and thus may not necessarily lead to immediate clinical complications. Various occupational activities involved in heating and burning of mercury are associated with elevated blood mercury. Hence the need for the use of personal protective equipment.

## Supporting information

S1 File Questionnaire for socio-demographic information, occupational activities and known clinical condition of the study participants. (PDF)
