# Supplementary material for: Variation in thyroid hormone levels is associated with elevated blood mercury levels among artisanal small-scale miners in Ghana
Source: PLoS One. 2018 Aug 30;13(8):e0203335. doi: 10.1371/journal.pone.0203335 (PMC6117084; doi:10.1371/journal.pone.0203335)
Supplement: S1 File — (PDF) [file pone.0203335.s001.pdf]

Questionnaire for socio-demographic information, occupational activities and known clinical condition of the study participants.

You have been asked to participate in this study which seeks to assess the thyroid hormone levels among artisanal small-scale miners as part of a research project conducted by Justice Afrifa, a staff member at the department of Medical Laboratory Science, University of Cape Coast. Your responses are entirely voluntary, and you may refuse to complete any part or all of this survey. We will protect information about you to the best of our ability. You will not be named in any reports. If you do not wish to answer any of the questions included in the survey, you may skip them and move on to the next question.

Subject Number............ Name of site...................... Date..........

A. Personnel Profile

A1. Sample ID [ ] A2. Age. **[ ]** A3. Gender: 1. Male [ ] 2. Female [ ]

A4 Educational Level: 1. None **[ ]** 2.Primary[ ] 3. Secondary [ ] 4.Tertiary [ ]

A5 Marital Status: 1. Single [ ] 2.Married [ ] 3. Divorced [ ] 4.Widowed[ ]

A6 Ethnicity…………………………………… A7 Nationality……………………………

B. Occupational Exposure and Safety

B1 How long have you been working as an informal miner? [ ]

B2 How many days do you work a week? [ ]

B3 How many hours do you spent per day at this mine [ ]

B4 Do you move between mines in a day? 1. Yes[ ] 2. No [ ]

B5 If yes, how many times 1. 2 times [ ] 2.3 times[ ] 3. 4 times[ ] 4. more than 4 times[ ]

B6 what do you usually do at this site?

(**Tick all that apply**)

1. Amalgamating with Mercury [ ]
2. Burning of Amalgam [ ]

3. Smelting of Gold [ ]

4. Transporting of Mercury [ ]

5. Transporting of ore [ ]

6. Standing in pool of water/stream whiles working [ ]

7. Sucking excess mercury from amalgam for reuse [ ]

B7 Which of the following personal protective equipment's do you use during the ore processing? **Circle all that applies**

	**Always**	**Seldom**	**Don't use**
1. Rubber aprons	1	2	3
2. Mercury containers	1	2	3
3. Face masks	1	2	3
4. Rubber gloves	1	2	3
5. Leather boots	1	2	3
6. Head coverings	1	2	3

B8 Have you worked at the goldsmith's shop before? 1.Yes [ ] 2. No [ ]

B9 If yes, for how many years [ ]

B10 where do you store the mercury? 1.Home [ ] 2. Mine site [ ] 3. Do not keep mercury [ ]

B11 Do you know the hazards in using mercury? 1.Yes [ ] 2. No [ ]

B12 Were you working at other mines before coming to Bibiani? 1.Yes [ ] 2.No[ ]

B13 Have you ever received any training on occupational safety and health? 1.Yes[ ] 2. No[ ]

B14 If yes, who provided it. (Circle all that apply)

1. Environmental Protection Agency [ ]
2. District Assembly [ ]
3. Small Scale Mining Association [ ]
4. Formal Mining Companies [ ]

5. Others (Specify)……………………………………………….

B15 What is your main source of drinking water at this mine?

1. Borehole [ ]
2. Pipe borne water [ ]
3. River/Stream [ ]
4. Sachet water [ ]

B16 Do consumes fish from the Amponsem lake or the river at Bibiani? (1) No (2) Yes

B17 Do you have any dental fillings? 1 No [ ] 2. Yes[ ]

C. Signs and Symptoms of Mercury Exposure

C1 Do you have any of these health problems (Circle all that applies)

	Yes	No
1. Skin rashes	1	2
2. Frequent cough	1	2
3. Persistent fever	1	2
4. Persistent headache	1	2
5. Metallic taste	1	2
6. Fatigue	1	2
7. Muscle aches	1	2
8. Numbness	1	2
9. Hair loss	1	2
10. Insomnia	1	2
11. Tremor	1	2

Others (specify) ……………………………………………………………..

C2 Have you been previously diagnosed of any of these diseases (Circle all that applies)

	Yes	NO	Do not Know
1. Hyperthyroidism	1	2	3

2. Hypothyroidism	1	2	3
3. Hashimoto's disease	1	2	3
4. Graves’ disease	1	2	3
5. Goiter	1	2	3
6. Thyroid nodules	1	2	3

7. Other (specify) ………………………………………………………………

Thank you for participation in this study
